# Bringing genomics to the field: An integrative approach to seed sourcing for forest restoration

**DOI:** 10.1002/aps3.11600

**Published:** 2024-06-20

**Authors:** Anoob Prakash, Thibaut Capblancq, Kathryn Shallows, David Saville, Deborah Landau, Chad Landress, Tal Jacobs, Stephen Keller

**Affiliations:** ^1^ Department of Plant Biology University of Vermont Burlington Vermont USA; ^2^ Laboratoire d'Écologie Alpine, Université Grenoble‐Alpes, Université Savoie Mont Blanc, CNRS Grenoble France; ^3^ Central Appalachians Program, The Nature Conservancy Elkins West Virginia USA; ^4^ Appalachian Forest Restoration LLC Morgantown West Virginia USA; ^5^ Maryland/DC Chapter, The Nature Conservancy Bethesda Maryland USA; ^6^ USDA Forest Service, Monongahela National Forest Elkins West Virginia USA; ^7^ Clinch Valley Program, The Nature Conservancy Abingdon Virginia USA

**Keywords:** admixture provenancing, conservation genomics, exome capture, genetic diversity, genetic load, *Picea rubens*, red spruce

## Abstract

**Premise:**

Global anthropogenic change threatens the health and productivity of forest ecosystems. Assisted migration and reforestation are tools to help mitigate these impacts. However, questions remain about how to approach sourcing seeds to ensure high establishment and future adaptability.

**Methods:**

Using exome‐capture sequencing, we demonstrate a computational approach to finding the best *n*‐sets from a candidate list of seed sources that collectively achieve high genetic diversity (GD) and minimal genetic load (GL), while also increasing evolvability in quantitative traits. The benefits of this three‐part strategy (diversity‐load‐evolvability) are to increase near‐term establishment success while also boosting evolutionary potential to respond to future stressors. Members of The Nature Conservancy and the Central Appalachian Spruce Restoration Initiative planted 58,000 seedlings across 255 acres. A subset of seedlings was monitored for establishment success and variation in growth.

**Results:**

The results show gains in GD relative to GL and increases in quantitative genetic variation in seedling growth for pooled vs. single‐source restoration. No single “super source” was observed across planting sites; rather, monitoring results demonstrate that pooling of multiple sources helps achieve higher GD:GL and evolvability.

**Discussion:**

Our study shows the potential for integrating genomics into local‐scale restoration and the importance of building partnerships between academic researchers and applied conservation managers.

Anthropogenic change in recent decades has caused widespread impacts on natural systems (Parmesan and Yohe, 2003), especially in forests, which function as a primary carbon sink for terrestrial ecosystems. Atmospheric warming has shifted the treelines towards high elevations and increased outbreaks of insect pests, while changes in precipitation have played a major role in increasing drought‐induced mortality (Pachauri et al., 2014). As a result, the abundance and extent of tree species are expected to decline due to the direct effects of higher temperature, drought stress, and/or failure to meet chilling requirements (Kimmins and Lavender, 1987; McCreary et al., 1990). Likewise, human changes in land use and deforestation have fragmented forests, reducing both the size and connectivity of remaining populations. These problems are compounded in long‐lived organisms like trees, which cannot keep pace with the changing climate through natural dispersal and whose long generation times slow the replenishment of genetic diversity lost due to mortality or demographic bottlenecks.

Faced with these challenges, forest restoration practitioners are increasingly seeking out approaches that can ensure near‐term establishment success while also increasing the capacity for restored populations to adaptively respond to future disturbance. Both goals benefit from a careful consideration of the seeds sourced for restoration. Traditionally, many restoration projects sought to source seeds or propagules from a local population in the immediate vicinity of the restoration site, with the rationale being to preserve local adaptation (the “local is best” paradigm). However, a limitation of this approach is potentially constraining the genetic diversity of the restoration propagule pool. Considering genetic diversity when sourcing seeds for restoration may be especially important for focal species occupying impacted landscapes, where the habitat has been degraded or population abundances reduced. Impacted landscapes often experience negative genetic consequences such as depleted genetic diversity due to fragmented and small populations (Eckert et al., 2008; Vranckx et al., 2012), inbreeding and the accumulation of mutation load (Willi et al., 2018; Hoffmann et al., 2021), and reduced gene flow through pollen and seed dispersal (Sork et al., 2002; Vakkari et al., 2006; Vranckx et al., 2012). In addition, decades of land use change and climate warming have already altered local ecosystems such that increasing the capacity of response to changing conditions might be more important than precisely matching historic or projected conditions (Pecl et al., 2017). Therefore, selecting seed sources/provenances with an eye towards capturing a representative fraction of the regional diversity is an important decision for restoration ecologists.

One way to achieve this is by integrating population genomic approaches with restoration planning to inform seed sourcing decisions. With the advancement of high‐throughput sequencing technologies, it has become increasingly feasible and cost‐effective to characterize diversity across the genome for a host of potential seed sources being considered for specific restoration sites. The challenge then becomes designing sampling strategies to best capture the diversity needed for short‐term establishment success and long‐term resilience in restored populations (Hoffmann et al., 2021). Based on core tenets of conservation genetics, the ideal seed pool for a restored population would possess high genetic diversity to avoid genetic drift and decrease the frequency of deleterious mutations, while also possessing sufficient variation in adaptive traits to enable evolutionary responses to future selection pressures (Willi et al., 2022). One strategy to establish this seed pool would be to sample multiple sources to capture more of the standing genetic variation available, an approach variously known as “mixed,” “composite,” or “admixture” provenancing (Prober et al., 2015; Bucharova et al., 2019; Hoffmann et al., 2021). The rationale is that single or local sources are unlikely to contain sufficient genetic diversity to avoid drift load, inbreeding, and reduced evolutionary potential (Nei et al., 1975; Leberg, 1992; Breed et al., 2019). By pooling multiple sources, diversity and resilience could be increased through a type of genetic “portfolio effect” (Schindler et al., 2015). An important consideration for admixture provenancing is the need to avoid outbreeding depression and the dilution of local adaptation that could arise if sources adaptively divergent from the target site are included. A balanced approach is then to restrict seed sourcing to sites within the same region as the target restoration site to limit the sampling to a common set of environmental conditions and biogeographic history—a strategy termed “regional admixture provenancing” (Bucharova et al., 2019).

As important as a diverse seed pool, close coordination between geneticists and restoration practitioners is critical to restoration success. Genomic‐assisted seed selection and restoration planning should not be a uni‐directional recommendation, but rather requires co‐creation of restoration goals, implementation, and monitoring of outcomes. Bringing genomics to the field for restoration should be an iterative feedback loop between the research and application partners, starting with (1) gathering genomic data and sharing insights on population diversity, demographic history, and adaptive variation across the range. (2) This information then forms the basis of discussions about defining the target region for considering potential seed sources for use in admixture provenancing. (3) Candidate sets of seed sources within that region are identified using genomic metrics to maximize the diversity profile. (4) These candidate sets are then vetted for access, feasibility, and cultural valuation for sampling and propagation. (5) Discussions of on‐site planting and design for future monitoring include field‐based experimental plots at the restoration sites that can be used to assess restoration success. (6) The restoration monitoring data are shared and discussed between the partners to identify and help fill the gaps in knowledge between basic science and field applications.

In this study, we describe a new approach to identifying potential seed sources for regional admixture provenancing based on maximizing available genetic diversity (GD) while minimizing the negative effects of genetic load (GL) due to drift. We demonstrate our approach using the restoration of fragmented red spruce forests in the central Appalachians of the eastern United States as a case study for integrating genomics with applied restoration and as an illustration of the co‐creation of successful partnerships between basic researchers and applied practitioners (Video S1).

## METHODS

### Study species

Red spruce (*Picea rubens* Sarg.) is a temperate coniferous tree species that has experienced widespread decline due to anthropogenic causes since the late 19th century (Reams and Deusen, 1993). It is also a prime example of a tree species vulnerable to rapid changes in climate due to its isolated populations and limited within‐population genetic diversity in the southern part of its range (Capblancq et al., 2020, 2021). In the central and southern Appalachians, populations are fragmented and largely limited to the slopes and mountaintops at elevations above 1066 m in West Virginia and above 1371 m in North Carolina and Tennessee (USDA, 2021). Red spruce lost the majority of its original extent in West Virginia and Virginia due to extensive logging and fire from the late 1800s to early 1900s that extirpated most of the original forests (Korstian, 1937) and later due to atmospheric pollution and acid deposition that resulted in further decline in the health and vigor of trees (Mathias and Thomas, 2018).

To employ a genomic‐guided approach to seed selection and monitor its influences on restoration success and functional trait variation, this study partners with the Central Appalachian Spruce Restoration Initiative (CASRI). CASRI members aim to restore red spruce ecosystems across high‐elevation landscapes of central Appalachia, using restoration planting and other silvicultural methods. Through these approaches, CASRI members work to expand and connect existing forest blocks of red spruce in the central Appalachians to enhance gene flow across the landscape and to ensure connectivity for spruce‐dependent species.

### Genomic variation

#### Exome‐capture sequencing and annotation

To profile the genomic diversity of potential seed sources for red spruce restoration, we used previously published whole‐exome capture sequencing of red spruce (Capblancq et al., 2020). The full data set consists of sequenced exomes (i.e., the protein‐coding gene space in the genome) for 339 individuals sampled from 65 populations across the range, with most populations represented by 5–6 individuals (Appendix S1). Because sequencing coverage was 2–3×, we used analysis of next‐generation sequencing data (ANGSD) (Korneliussen et al., 2014) to produce genotype likelihoods for each individual after quality control filtering (see Capblancq et al., 2020 for specific filtering criteria). To annotate genetic variants based on functional classes, we used SnpEff v5.1 (Cingolani et al., 2012) based on the Norway spruce (*Picea abies* (L.) H. Karst) (Nystedt et al., 2013) genome annotation v1.0. These data were then used to estimate measures of genomic variation for each population and for combinations of populations that might serve as candidate seed sources (see below).

#### Genetic diversity and genetic load estimation

We focused on two measures of genomic variation to evaluate candidate sources—GD and GL. These two measures were chosen based on our previous work in red spruce, in which they were found to be significant predictors of early‐life fitness of seedlings (Capblancq et al., 2021). GD was estimated based on expected heterozygosity (*H*_e_) across all single‐nucleotide polymorphisms (SNPs), which provides a fundamental measure of genetic variation based on the frequency of heterozygous genotypes expected under Hardy–Weinberg equilibrium (Nei, 1973). GD is lower in small or bottlenecked populations experiencing genetic drift, resulting in reduced effective population size (*N*_e_) and adaptive potential compared to large populations (Allendorf, 1986). GL measures the accumulation of deleterious mutations in the population due to GD (i.e., drift load). To obtain an estimate of GL in populations, we calculated the number of non‐synonymous (amino acid–changing) SNPs (Pn) and the number of synonymous (silent) SNPs (Ps), weighted by their corresponding frequencies (fn and fs, respectively). We then estimated GL as the ratio Pnfn/Psfs under the well‐supported assumption that the majority of nonsynonymous mutations are deleterious (Willi et al., 2018). We collectively treated the following functional categories from SnpEff as non‐synonymous in our GL calculation: *missense variant*, *splice acceptor variant*, *splice donor variant*, *splice region variant*, *start lost*, *stop gained*, *stop lost*. GD and GL were then estimated for different source combinations for each restoration site using the *combn* function of the R package R.utils v2.12.3 (Bengtsson, 2023) incorporated into a custom function *optimize* (see Data Availability Statement for code used for genomic source selection and statistical analysis). There was no correlation between GD and GL for the sources tested (Pearson's correlation: −0.135, *P* value = 0.56).

### Seed source selection model

#### Defining the regional focus

The regional admixture provenancing strategy is based on sourcing seeds from a set of populations within the region of the target restoration sites that share a common biogeographic history and patterns of local adaptation (Bucharova et al., 2019). The targeted restoration sites in Maryland (MD), Virginia (VA), and West Virginia (WV) are within red spruce's southern range edge, which consists of a regional genetic ancestry group that split from the rest of the range ca. 8000 years ago (Capblancq et al., 2020). Populations within this region also occupy unique climatic environments and show trait divergence from more northern populations based on common garden studies (Prakash et al., 2022), suggesting regional‐scale local adaptation to environmental conditions within the central and southern Appalachians (Prakash and Keller, unpublished data). Based on the unique evolutionary history and local adaptation of this region, we restricted our search for candidate seed sources to the 23 southern range edge populations of Capblancq et al. (2020). To further refine our selection of potential sources based on qualitative assessment, we utilized a combination of geographical proximity and expert opinion within our group. This approach was taken to minimize the risk of disrupting local adaptation. We selected sources from within the same state in cases where we identified 10 or more potential sources, as we did for the WV site. When fewer than 10 sources were available, we expanded our selection to include sources from adjacent states to meet this threshold. In addition, two populations were removed because they showed northern ancestry due to previous transplantation (i.e., XCV and HR; Capblancq et al., 2020).

#### Optimizing seed source combinations

**Figure 1:**
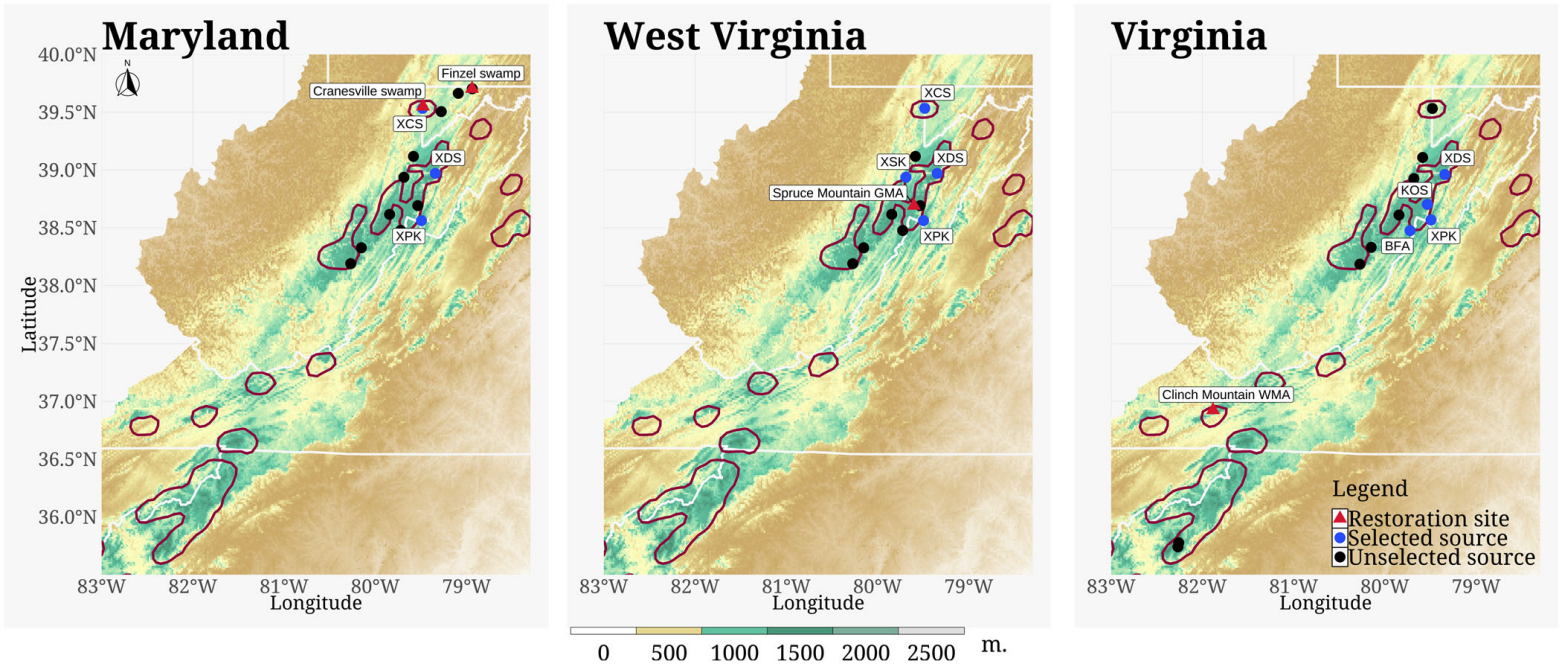
The location of red spruce restoration sites and the seed sources selected for planting sites in Maryland, West Virginia, and Virginia. The dark red outlines on the map show the known range extent of red spruce (Little Jr., 1971).

Seed sources were selected based on optimizing the ratio of GD to GL in sets of *n* source populations from within the southern range edge. We focused on GD:GL because previous work suggested each component of genomic variation was associated with early‐life fitness (Capblancq et al., 2021), and a reanalysis of these data for just southern edge populations confirmed these associations (Appendix S2). We determined the GD:GL for a combined sample set, consisting of three to four sources selected from a regional pool of potential sources geographically proximate to each target site (Figure 1). In this way, we sought out combinations of sources that together achieved high pooled diversity while minimizing pooled load (e.g., high GD:GL), even if diversity or load within any single source was suboptimal. Candidate sets of *n* sources were then presented to conservation partners at CASRI for feedback and consideration of factors such as collection logistics and seed crop availability. This led to the elimination of certain high‐ranking sets based purely on GD:GL criteria but enabled more flexibility for seed collection work.

### Restoration planting

#### Site selection and seedling propagation

The Nature Conservancy (TNC), a long‐time CASRI member, procured funding in 2019 from the Wildlife Conservation Society's Climate Adaptation Fund for red spruce restoration on 255 acres on both private and public lands within TNC's focal landscapes in MD, VA, and WV. The specific sites were TNC's Finzel Swamp Preserve in MD (39.703°N, −78.940°W), TNC's Cranesville Swamp Preserve in MD (39.550°N, −79.479°W), TNC's Rich Mountain Farm forest management easement and the adjacent Clinch Mountain Wildlife Management Area in VA (36.925°N, −81.879°W), and Spruce Mountain Grouse Management Area on the Monongahela National Forest in WV (38.691°N, −79.599°W) (Figure 1). All restoration sites are within the historic range of red spruce, but populations had been significantly reduced by unsustainable logging. Overall, the sites were chosen to expand existing stands and create greater landscape connectivity where possible.

The project sites in MD are both “frost pockets,” with surrounding mountains funneling cooler air into these low bowls, resulting in consistently cooler temperatures. These sites are low, wet, and have species assemblages reminiscent of habitats much farther north in Canada that are remnants from the Pleistocene Epoch. The Finzel Swamp (845 m elevation) planting sites were adjacent to a boreal fen, in open areas that had been farmed and grazed until the land was sold to TNC in 1970. The Cranesville Swamp (790 m elevation) site was adjacent to a boreal peat bog and had been managed as a hay field up until 2014. A few historic stands of red spruce remain at both these sites, adjacent to or within the wetlands.

In WV, the project sites are open riparian areas (1133–1234 m elevation) that remained unforested due to past logging operations, subsequent livestock grazing, and competition from herbaceous vegetation. The sites were selected as important high‐elevation stream corridors to provide shading for the cold‐water ecosystem and enhance the connectivity of larger blocks of spruce forests to the east and west. Planting avoided areas that were currently or may be influenced by beaver activity and saturated soils. In VA, the project sites were selected to expand the current red spruce population and to increase connectivity across the highest elevations (~1200 m) adjacent to areas of natural regeneration and little competition from hardwood regeneration. Planting was limited to accessible slopes and soil conditions suitable for planting. Out of the 23 seed sources considered (see above), we limited the sources to those regionally proximal to the specific restoration site (Figure 1). Out of the considered populations, *N* = 6 were selected for planting overall, although not all sources were planted at each site—a unique three‐source set was used for the two MD sites, a unique four‐source set for the WV site, and an additional unique four‐source set for the VA site (Table 1).

**Table 1:** Details regarding the red spruce seed sources, seedlings planted at each site, and number of experimental plots at each restoration site.

Restoration site	No. of seedlings planted						No. of sources	Total no. of experimental plots
	Cranesville Swamp, WV (XCS)	Panther Knob, WV (XPK)	Dolly Sods, WV (XDS)	Stuart Knob, WV (XSK)	Camp Allegheny Battlefield, WV (BFA)	Spruce Knob, WV (KOS)		
Virginia (VA)	–	200	200	–	200	200	4	20
West Virginia (WV)	200	200	200	200	–	–	4	20
Maryland (MD)	240	240	240	–	–	–	3	9 × 2 = 18

A cone crop in autumn 2019 allowed us to make collections from the six targeted sources. Seeds were collected within 1–2 acres of these targeted sources, and each source consisted of 20–40 mother trees. Cones were collected, cured, and kiln dried, and the seed was extracted and de‐winged. To produce seedlings within the timeframe necessary, germination testing was not performed, and instead the seed was purified as well as possible using a simple cut test to determine viability. Itasca Greenhouse in Cohasset, Minnesota, germinated the seed in winter 2020 and grew seedlings in 164‐cm^3^ plugs in climate‐controlled greenhouses. In spring 2021, contractors planted 58,000 red spruce trees derived from these selected seed sources, along with 32,000 associated native trees and shrubs selected to achieve broader restoration habitat goals at each site. The numbers of seedlings planted at each site by seed source are detailed in Table 1, and the climate space occupied by the restoration sites are shown with a climate principal component analysis (PCA) in Appendix S3.

#### Monitoring survivorship and height growth

**Figure 2:**
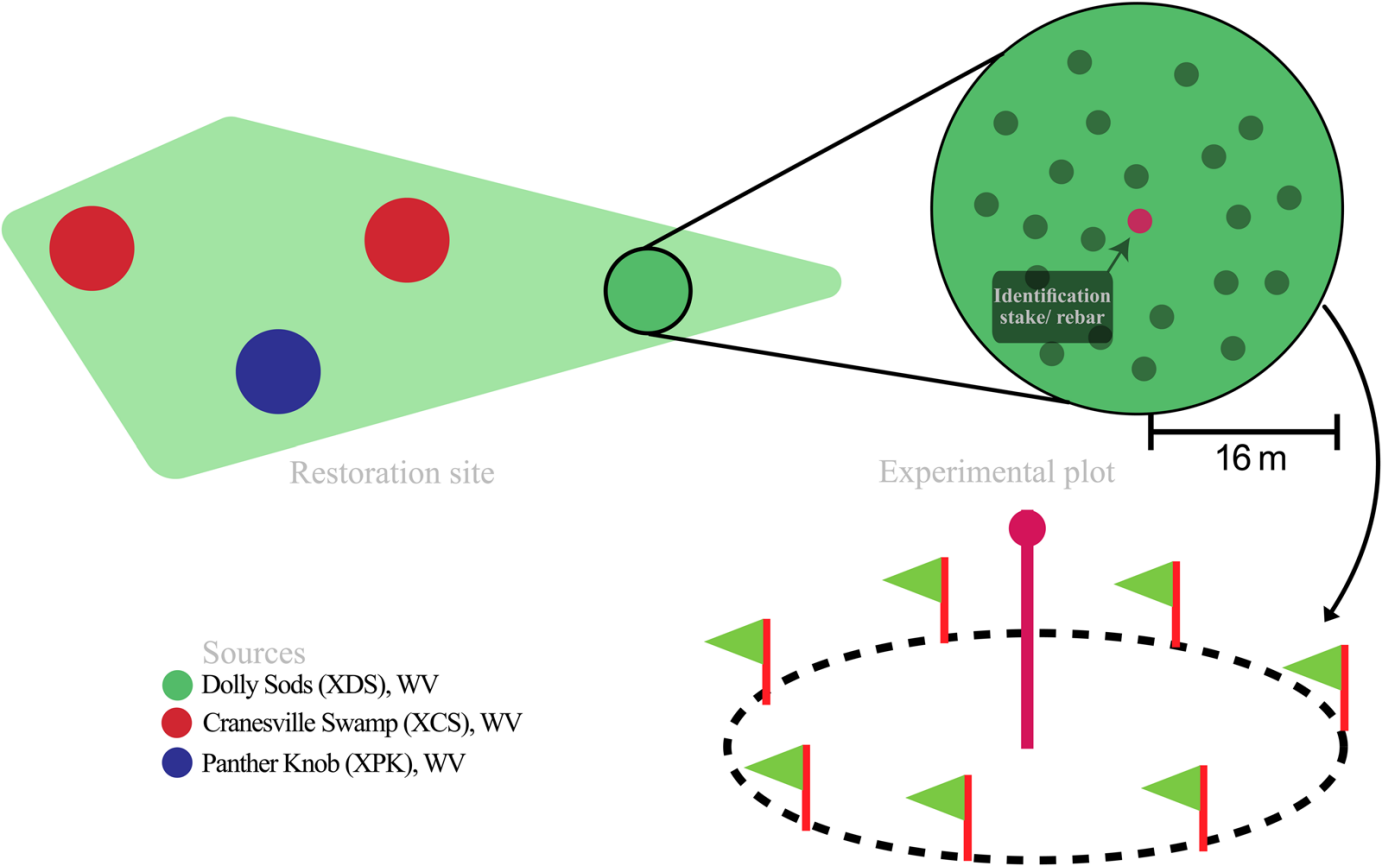
Diagram illustrating the experimental design for the red spruce monitoring effort. A circular experimental plot with a radius of 16 m was established at the restoration site for reforestation monitoring and to assess the performance of individual sources. Each experimental plot consisted of 40 plants from a single source to reflect the planting density of the reforestation activity. These single‐source experimental plots were marked with a central metal rod with a bright orange cap and identified with GPS coordinates for future monitoring efforts.

We initiated a monitoring protocol at planting to enable tracking of establishment success (survival and growth through the second year). The monitoring design consisted of replicate georeferenced experimental plots stratified by seed source so that we could assess single‐source variability at each site and compare the effect of single vs. pooled sources on trait variance (Figure 2). The locations of experimental plots were intermixed within the boundaries of each restoration site, with positions chosen randomly using the *create random points* function in ArcGIS v2.6.0 (ESRI, 2011). Plot locations were buffered by 18 m (16‐m plot radius + 2‐m buffer) in ArcGIS to avoid overlapping adjacent plots. Assigned plot positions were then ground‐truthed by the site leads, and when necessary, adjusted in location to avoid overly steep slopes, excessive soil saturation, and evidence of potential impacts from current or future beaver activity.

Each source was planted in five replicate plots within each site in spring 2021. For each plot, 40 seedlings from the designated source were planted randomly within a 16‐m radius of the plot center to achieve a planting density of 0.05 plants/m^2^ (Figure 2), which was representative of the planting density outside the plots. In total, we established 20 plots at the VA and WV sites and 18 plots at the MD sites (nine plots each at Cranesville Swamp and Finzel Swamp). The total number of research seedlings at VA and WV at the time of planting was 800 trees per site (4 sources × 5 plots × 40 plants), and at MD was 720 trees (3 sources × 3 plots × 2 sites × 40 plants), or *N* = 2320 total research seedlings monitored (Table 1).

Data on establishment success were collected one year after planting at the beginning of the growing season (April 2022). All plants within the experimental plots were located, tagged, and measured for seedling height (centimeters from soil surface). Survivorship was also estimated as the proportion of alive plants out of the 40 initially planted within each plot.

#### Statistical analyses

Height and survivorship data were analyzed for variance among sources using linear mixed‐effects models implemented with the lme4 package (Bates et al., 2015), with source as a fixed effect and plots within each site as a random effect. Plant height was modeled with a Gaussian distribution and survivorship with a binomial distribution.

We were also interested in analyzing the effect of single vs. pooled sources on the evolutionary potential of trait variance in the restored populations, with the expectation that pooling sources together increases genetic variance in adaptive traits, thus widening the genetic pool for selection to act on in the future under environmental change. To do this, we used the seedling height data to calculate a scale‐free measure of evolvability based on the genetic coefficient of variation (Houle, 1992) (Equation 1):

(1) $$\text{Coefficient of evolvability},\;{\mathrm{CV}}_{G}=\frac{\surd V_{G}}{\bar{X}}$$

Where *V*_G_ is the genetic component of trait variance, and $\overset{®}{X}$ is the trait mean. In this study, we did not know the pedigree of restoration seedlings, so we estimated *V*_G_ by multiplying the total phenotypic variance (*V*_P_) by an estimate of the broad‐sense heritability (*H* ^2^ = *V*_G_/*V*_P_) of one‐year seedling height from a previous common garden field experiment (Prakash et al., 2022) subsetted for only southern edge populations (*N* = 110 families across 23 populations) planted at the common garden site in Frostburg, Maryland. Variance components were estimated with a Bayesian approach in the R package MCMCglmm (Hadfield, 2010) for an *H* ^2^ estimate of 0.3962. From this, we obtained an estimate of *V*_G_, and evolvability was calculated using the modeled heights from the experimental plots after removing the outliers based on the interquartile range (Tukey, 1977). In order to account for the sample size differences between the *CV*_G_ of individual sources and the *CV*_G_ for the pooled sources within a restoration site, the pooled *CV*_G_ was bootstrapped 1000 times based on random sampling with replacement of *N* = 200 individuals (to reflect the individual source *N* at each site, in the absence of mortality). The mean and standard deviation of this bootstrapped *CV*_G_ was then used to represent the pooled *CV*_G_. In addition, we estimated *CV*_G_ for phenological traits (bud break and bud set) measured during the common garden experiment conducted by Prakash et al. (2022) to compare evolvability of different traits during the early establishment phase of red spruce seedlings. The phenological trait variance was measured on all the southern edge populations grown in the MD common garden site.

## RESULTS

### Genetic profile of seed source combinations

**Figure 3:**
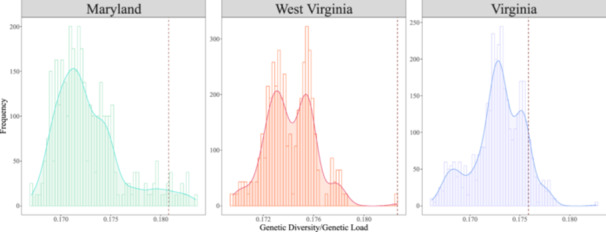
Genetic diversity:genetic load (GD:GL) for the source combinations selected for each restoration site. The dotted red vertical line indicates where the selected source combinations fall for the respective restoration site. At the Maryland site, three‐source combinations were used, while the West Virgina and Virginia sites had four‐source combinations out of the 23 total sources available at the southern range edge of red spruce.

GD and GL were variable across all possible sets of source populations, suggesting that source selection would influence GD:GL in the restoration seed pool. The sets of *n* = 3 source populations considered for the MD site showed a GD range between 0.170 and 0.177 and a GL range between 0.955 and 1.043, with the ratio of GD:GL ranging from 0.167 to 0.184 (mean = 0.173) (Figure 3). Similar values were observed among the sets of *n* = 4 source populations at the WV site (GD: 0.175–0.180, GL: 0.962–1.044), with GD:GL ranging from 0.175–0.183 (mean = 0.174). Finally, for the VA site, variation among the sets of *n* = 4 source populations was similar to the WV sets (GD: 0.174–0.180, GL: 0.962–1.053), with the ratio of GD:GL ranging from 0.166–0.182 (mean = 0.173).

After reviewing the best *n* sets for each restoration site and considering logistics of access and cone crop availability, the sources selected for the MD site were XCS (Cranesville Swamp, WV), XDS (Dolly Sods, WV), and XPK (Panther Knob, WV) (Figure 1). For the WV site, the sources selected were XCS, XDS, XPK, and XSK (Stuart Knob, WV). Finally, for the VA site, the sources selected were KOS (Spruce Knob, WV), BFA (Camp Allegheny Battlefield, WV), XDS, and XPK. The three‐source combination XCS‐XDS‐XPK (GD = 0.174, GL = 0.961, GD:GL = 0.181) was in the 96th percentile of GD:GL source combinations considered for MD (Figure 3). The four‐source combination XCS‐XDS‐XPK‐XSK (GD = 0.176, GL = 0.962, GD:GL = 0.183) was the best (i.e., 100th percentile) source combination for the WV site. Finally, the source combination BFA‐KOS‐XDS‐XPK (GD = 0.178, GL = 1.011, GD:GL = 0.176) was in the 91st percentile of GD:GL estimate for the VA site.

### Field monitoring of survivorship and height

**Figure 4:**
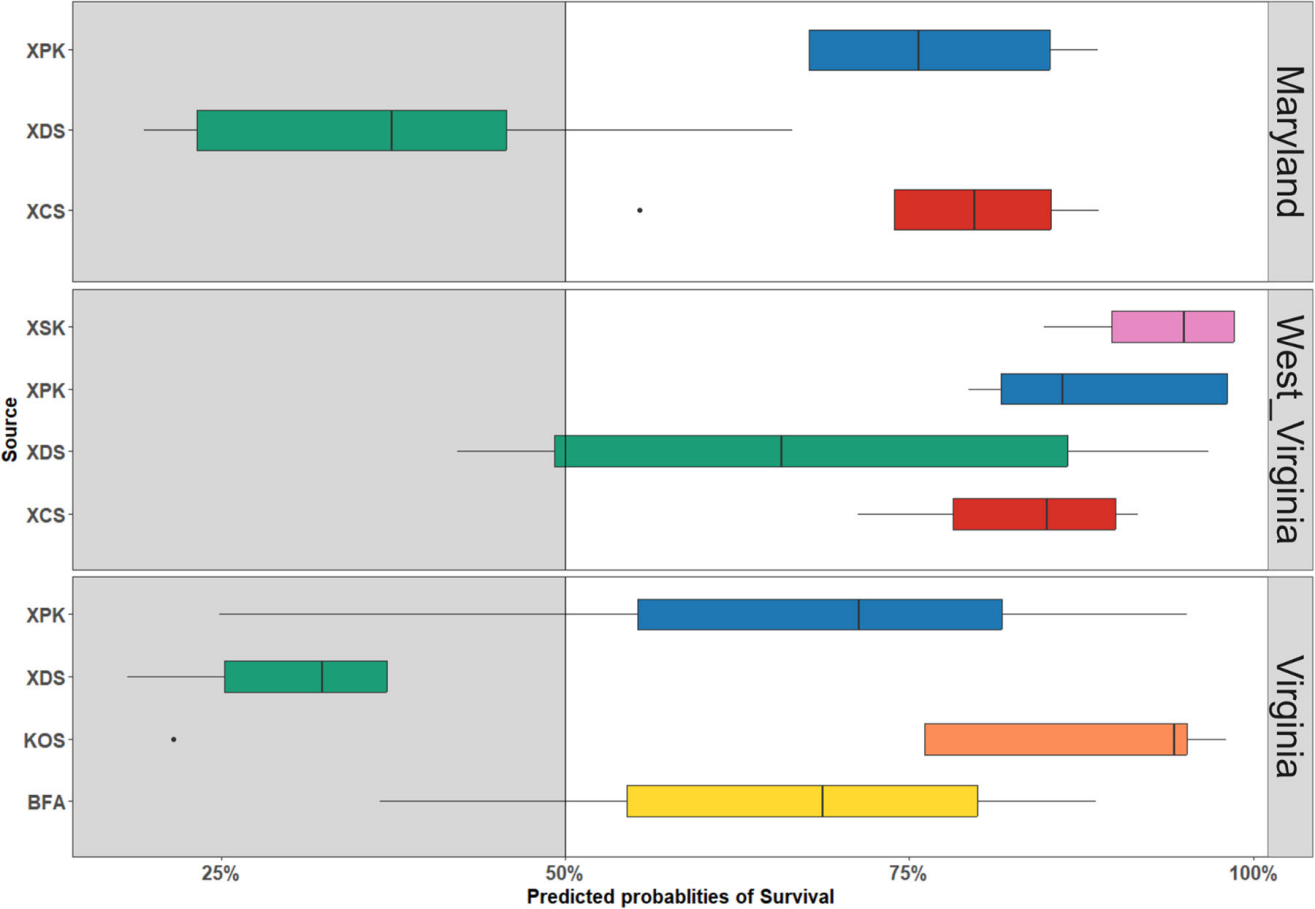
Survivorship of individual sources at each restoration site one year after planting. See Table 1 for source abbreviations.

Survivorship after one year at the MD site showed significant variation among sources (Figure 4), with XDS seedlings experiencing significantly lower survival than XCS (*P* < 0.001) or XPK (*P* < 0.001). At the WV site, there were no significant differences in survivorship among sources, although XCS again had the highest survivorship and XDS the lowest survivorship. At the VA site, XDS again showed the lowest survivorship, differing significantly from KOS (*P* < 0.05). For individual source survivorship across multiple sites, XDS exhibited significantly higher survivorship at WV compared to the MD (*P* < 0.05) and VA (*P* < 0.05) sites. However, there were no significant differences between survivorship for XPK and XCS across sites.

**Figure 5:**
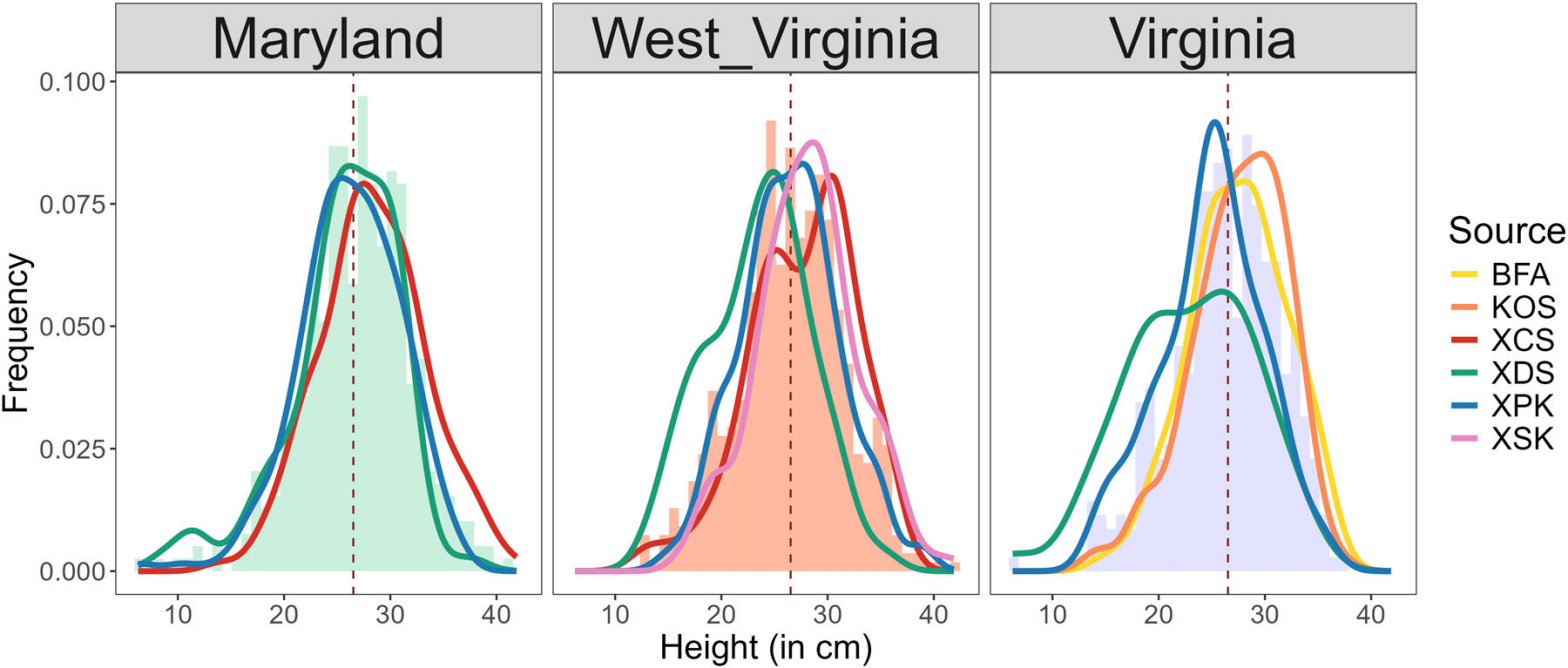
Modeled height at each restoration site. The colored lines denote the distribution of seedling height per source, and the histogram shows the distribution of the pooled height at each site. The dotted vertical line passes through the mean of the pooled height for each site. See Table 1 for source abbreviations.

The height of seedlings at the start of their second year varied significantly among single sources, being higher for XCS compared to XDS (*P* < 0.01) (Figure 5). Similarly, at the WV site, differences in height were again significant among sources, driven by the lower height of XDS compared to all three other sources (XSK, XCS, and XPK; all *P* < 0.0001). The results were similar at the VA site, with differences among sources driven by the lower height of XDS compared to KOS (*P* < 0.001, 4.90 cm) and BFA (*P* < 0.001, 4.83 cm). For sources planted across at least two restoration sites, the sources XCS, XDS, and XPK did not show any significant differences in height across the three restoration sites.

### Evolutionary potential of the seed sources

**Figure 6:**
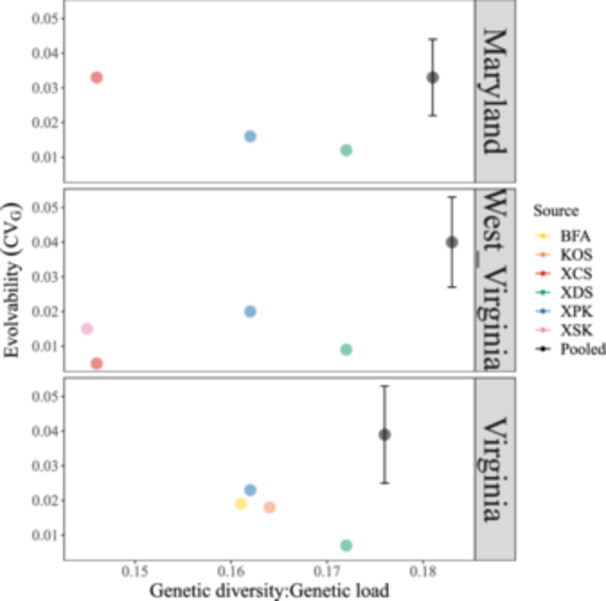
Evolvability (*CV*_G_) for different sources planted at each restoration site, and the bootstrapped pooled *CV*_G_ for source combinations at each site. The *x*‐axis shows the GD:GL estimates for the singular and pooled sources. See Table 1 for source abbreviations.

In MD, the evolvability of one‐year seedling height was up to 2.8× higher for the combined restoration pool (*CV*_G_ = 0.033 ± 0.011) compared to individual sources (XCS, *CV*_G_ = 0.033; XDS, *CV*_G_ = 0.012; XPK, *CV*_G_ = 0.016) (Table 2, Figure 6). At the WV site, the evolvability of the restoration pool (*CV*_G_ = 0.04 ± 0.013) was up to 8× higher than individual sources (XCS, *CV*_G_ = 0.005; XDS, *CV*_G_ = 0.009; XPK, *CV*_G_ = 0.02; XSK, *CV*_G_ = 0.015). Finally, at the VA site, the evolvability for the restoration pool (*CV*_G_ = 0.039 ± 0.014) was up to 5.6× greater than individual sources (BFA, *CV*_G_ = 0.019; KOS, *CV*_G_ = 0.018; XDS, *CV*_G_ = 0.007; XPK, *CV*_G_ = 0.023). At all the restoration sites, the evolvability was highest for the combined pool rather than a single source, with only XCS having the same evolvability as the combined pool at its native site in MD.

**Table 2:** Evolvability (*CV*_G_) for one year height (in cm) at each restoration site. Sources with the highest evolvability in each restoration site are represented by gray‐shaded cells. The broad sense heritability (*H* ^2^) for height in red spruce is 0.396.

Region	Source	$\overset{®}{X}$	*V*_P_	*V*_G_	*CV*_G_
Maryland	XCS	28.0817	2.164	0.857	0.033
	XDS	25.652	0.247	0.098	0.012
	XPK	26.210	0.423	0.168	0.016
	Pooled	26.335	0.908	0.359	0.033 ± 0.011
West Virginia	XCS	27.273	0.055	0.022	0.005
	XDS	23.947	0.119	0.047	0.009
	XPK	26.489	0.729	0.289	0.02
	XSK	28.040	0.444	0.176	0.015
	Pooled	26.493	3.162	1.252	0.04 ± 0.013
Virginia	BFA	27.338	0.679	0.269	0.019
	KOS	27.228	0.582	0.231	0.018
	XDS	22.057	0.054	0.021	0.007
	XPK	25.052	0.870	0.344	0.023
	Pooled	26.047	3.801	1.505	0.039 ± 0.014

*Note*: $\overset{®}{X}$ =phenotypic mean,V_P_ = phenotypic variance, *V*_G_ = genetic variance, *CV*_G_ = coefficient of evolvability.

While we only measured a single trait (height) for evolvability in restored populations, for comparison with other traits, we also report broad sense heritabilities and *CV*_G_ values for southern edge populations measured in our previous common garden experiment in MD (Prakash et al., 2022). Phenological traits had the highest *H* ^2^ for bud set at the end of year 1 (*H* ^2^ = 0.1677) and lowest for bud set at the end of year 2 (*H* ^2^ = 0.017) (Appendix S4). Bud break had an *H* ^2^ estimate of 0.0274, and height at the end of year 1 had an *H* ^2^ estimate of 0.3962. To put in perspective the variability of *H* ^2^ measured across contrasting environments, the *H* ^2^ estimates for height in other common gardens were 0.2844 in Vermont (northernmost garden) and 0.3251 in North Carolina (southernmost garden). Evolvability for the common garden seedings was observed to be the highest for height at the end of year 1 (*CV*_G_ = 0.0581) out of all the traits studied (Appendix S4). The lowest values of evolvability were observed for bud set at the end of year 1 (*CV*_G_ = 0.0066) and year 2 (*CV*_G_ = 0.0006). Bud break had an evolvability value of *CV*_G_ = 0.001. The order of magnitude of evolvability is different from *H* ^2^ estimates, with bud break having higher evolvability than bud set. However, height remained the highest in both estimates out of all the common garden traits studied.

## DISCUSSION

### Genomics‐assisted restoration

As genomics has become more accessible for studies of non‐model organisms, it is increasingly being integrated into conservation planning and restoration of impacted species (Willi et al., 2022). A key contribution that genomics can offer conservation planning is guidance on how best to maintain and even enhance levels of GD within populations. This has been central to the approach we develop here with red spruce. Habitat fragmentation and anthropogenic disturbance have been found to contribute to genetic erosion, i.e., the loss of GD due to inbreeding and genetic drift (Woodruff, 2001), and reduce the adaptive responses of populations experiencing environmental stress (Brubaker, 1986; Frankham et al., 1999; Bijlsma and Loeschcke, 2012; González et al., 2020). In that context, GD is recognized as an important element of conservation planning and policy implementation (Hoban et al., 2021).

However, the scientific community is divided on the use of GD for conservation and the value of selectively neutral (or genome‐wide) GD estimates vs. GD at functional loci (Ralls et al., 2020; García‐Dorado and Caballero, 2021; Teixeira and Huber, 2021). One argument posits that neutral GD has a minor role in predicting species viability or extinction risks and suggests that functional GD, demographic history, and ecological relationships hold more information for species conservation (Teixeira and Huber, 2021). Another argument proposes that non‐genetic factors including habitat destruction, fragmentation, over‐harvesting, climate change, pollution, and introduction of invasive species are more important factors for population decline (Foden et al., 2019). Other researchers, however, have found strong benefits of using GD in conservation (Reed and Frankham, 2003; Ørsted et al., 2019). Indeed, key findings from conservation genetics in the past decade point to the importance of genome‐wide estimates of GD in conservation and restoration, with low GD associated with demographic bottlenecks and/or inbreeding depression that is linked to lower performance, reduced response to selection, and increased susceptibility to drift load (Willi et al., 2022).

Our work in red spruce supports the case for GD being a useful component of conservation and restoration work. In the fragmented southern range edge of red spruce, where habitat loss and demographic bottlenecks have been severe, we found a strong positive association between GD and early‐life fitness in red spruce, as well as an inverse association between early‐life fitness and GL (Appendix S2). Because these associations were most profound in the southern range edge (Capblancq et al., 2021; also refer to the Methods section, under the subsection “Optimizing seed source combinations”), this supports the importance of GD and GL to the populations that are most at risk and in need of restoration. The relevance of GD and GL to performance in range‐edge populations of red spruce is also informed by our knowledge of the longer‐term demographic history of this region. Capblancq et al. (2020) discovered red spruce has undergone a long‐term decline in *N*_e_ dating thousands of years, well before anthropogenic declines became important. Furthermore, the splitting of GD into three regional groups of genetic ancestry has left the southern range edge isolated and with the lowest *N*_e_ of the three regional groups, with *N*_e_ estimates ranging from 3000–11,000 (Capblancq et al., 2020). This is close to the minimum *N*_e_ recommended by conservation genetic principles to sustain healthy populations in the long term (≥1000 effective individuals; Frankham et al., 2014; Hoban et al., 2021). Therefore, the careful management of the diversity present within the southern range edge and its integration into restoration plantings takes on applied importance.

We found that taking a combinatorial approach to identify optimized sets of *n* seed source populations produces a restoration pool that, based on conservation genetic principles, should enhance the viability and resilience of the restored populations compared to sourcing seed from a single provenance. This assessment was based on two metrics: (1) a high GD:GL ratio from genomic data, which should increase the *N*_e_ of the population while minimizing the input of deleterious alleles, and (2) high trait evolvability (here, based on *CV*_G_ for height), used as an estimate of the evolutionary potential of the seed sources selected for each site. Across the sites, *CV*_G_ and GD:GL were highest for the pooled sources compared to any single source (Figure 6). High *CV*_G_ for functional traits such as height can increase the resilience of populations to future environmental perturbations (such as climate change) by increasing the population's genetic variability for traits to respond to novel selection pressures. We use *CV*_G_ as a proxy to measure evolutionary potential for traits measured in the field, not just height. The reason for pooling of sources is to increase the evolvability of as many traits as possible in the field. Even though height was the only trait measurable in the field to estimate *CV*_G_, it was also observed to be the trait with the highest *CV*_G_ in a controlled common garden environment compared to phenological traits like bud break and bud set (Appendix S4). Measuring these phenological traits in the field was logistically impossible, but *CV*_G_ measured in the common garden experiments helps to contextualize these values for red spruce seedlings of similar development phase growing in the natural environment.

There are both short‐ and longer‐term benefits to evolvability. Even though evolvability is measured in current generation seedlings, it provides an estimate of the capacity for within‐generation selection to produce a shift in trait mean. Ultimately, however, the longer‐term benefit of the regional admixture strategy will likely manifest from the offspring these restoration plantings produce after growing to reproductive age. These offspring will then mate with each other, recombining their genetic variation, which should further increase their adaptive potential to respond to a changing environment. This is difficult to observe for long‐lived organisms like red spruce, which take 30–40 years to produce seeds (Schopmeyer, 1974) and probably another 30 years for us to observe the ecosystem effects. This is one reason why research into seed sourcing for organisms with long generation times is challenging, in which the generation time of the organism may exceed the velocity of climate change. Thus, metrics such as optimizing diversity while minimizing load (GD:GL) and increasing evolvability can serve as useful guidelines for seed sourcing, but it must also be acknowledged that multiple approaches exist for integrating genomics for the restoration of long‐lived species. As such, it is important to report seed sourcing strategies in detail to provide future scientists and practitioners with the necessary information and tools to better assess the effectiveness of restoration methodologies employed to improve genetic diversity and trait evolvability (Jordan et al., 2019).

### Field performance of single vs. pooled seed sources

Enrichment planting has been recommended for red spruce as a way to revegetate scarce forest patches (Raymond and Bédard, 2017; Dumais et al., 2019). However, prior to our study, restoration planting of red spruce seedlings in the central Appalachians did not take into account the genetic diversity or source provenance of the seed. Thus, it was of interest to understand how the performance (survival and height growth) varied among the individual sources in situations such as ours where provenance is traceable, and whether certain sources stand out as “superior” across sites. The survival and growth of individual sources varied significantly within and between sites (Figures 4 and 5). For example, XDS (“Dolly Sods”) generally had the lowest survivorship, which was surprising given that seed from Dolly Sods has been frequently used for past restoration plantings of red spruce (D. Saville, personal observation). It remains to be seen if this was a localized effect (i.e., the specific locale or individual trees collected from, or a poor seed year) or a more general reflection of Dolly Sods as a source provenance for red spruce. We also note that XDS often achieved lower heights than other sources, which contributed to raising evolvability for this trait (Figure 6). Seedling heights were generally observed to have a mean height above 25 cm for most sources planted (Figure 5). There were no sources that seemed to outperform others in terms of pure growth potential, even though they exhibited variances in the distribution of the total height achieved after a year in the ground. Collectively, these insights serve as two important reminders—first, that source provenances do vary in survival and growth, with no one “super source” that is better than all other sources across sites; and second, slower growth may not necessarily signal poor performance, as we do not know what other functional traits may covary in these seedlings (for example, slow growth may come with increased stress tolerance).

It is also important to recognize that many non‐genetic ecological sources undoubtedly contribute to seedling performance. For example, performance of red spruce seedlings in the wild is known to suffer due to competing vegetation (Dumais and Prévost, 2007), slow development (Dumais and Prévost, 2016), shade requirement in early stages of development (Blum, 1990; Wu et al., 1999), and browsing of newly planted seedlings by animals because of their nutritional reserve (Burney and Jacobs, 2011). Browsing can also result in growth delays, deformation, and mortality (Gill, 1992). The survivorship for most sources hovered above 75% (Figure 4) after the first year of growth, which is promising as a considerable amount of the seedlings were planted in open forest patches along with associated native trees and shrubs. The VA sites had the lowest mean survival rate, probably due to exposure to desiccating winds and the southwest aspect of the planting area. There were also differences in the vegetation communities across the sites that may have affected seedling performance. We observed a high density of goldenrod (*Solidago canadensis* L.) at the MD and WV sites, which may have benefited the spruce seedlings by providing cover during the peak summer heat while allowing ample light during the spring and fall seasons, similar to seasonal light conditions under a hardwood forest canopy (Dumais et al., 2019). However, goldenrod could also have a competitive effect on seedling growth after initial establishment, so their long‐term effect is yet to be determined.

### Collaboration between science and practice: Lessons learned and a path to the future

The four foundational principles guiding restoration practices are to increase ecological integrity, to achieve long‐term sustainability, to be informed by the past and wary of the future, and to benefit and engage society (Suding et al., 2015). In keeping with these foundational practices of restoration, CASRI partners have implemented large‐scale efforts as well as planned for the future to ensure that red spruce is not limited to isolated high‐altitude stands at the southern range edge; in doing so, they have advanced a path of resilience against adversity in the face of human land‐use change and climate warming (Video S1). CASRI has been progressive in utilizing the latest scientific methodologies and research results to assist with conservation efforts and integrate new innovations into restoration practices, providing a potential path forward for successful partnerships between scientists and practitioners (Brancalion and van Melis, 2017; Breed et al., 2019; Willi et al., 2022; Theissinger et al., 2023). While conservation genetic guidelines for planning genetic rescue have been in place for some time, including the need to maximize the genetic diversity of fragmented populations while minimizing inbreeding and outbreeding depression (Frankham et al., 2017), these concepts and the data and interpretative guidance are not always accessible to restoration practitioners. Indeed, while CASRI members have been working to restore red spruce for years through experimental plantings and silvicultural treatments, this is the first time that seed sources were selected based on genetic information. In our partnership, we brought together restoration practitioners who have an intimate knowledge of the history of the landscape, an understanding of the vulnerability of the species, and knowledge of the applied principles of restoration to re‐establish plantings, with basic research scientists who offered new perspectives from genomics to guide seed selection using a regional admixture provenancing strategy to establish healthy and resilient populations.

Using multiple sources for restoration is not a new idea, and the collection of seeds from a region is an established practice in forest trees (Kitzmiller, 1990; Hufford and Mazer, 2003). Such regional admixtures are carried out in forestry, but the use of genomic data to identify source combinations within these regions, as done in our study, is rare. Most regional provenancing done in forest trees tends to be based on “seed transfer zones” that use one or more environmental characteristics shared between the seed collection and restoration sites so as to minimize the negative effect on population fitness (Kitzmiller, 1990; Hufford and Mazer, 2003; Bucharova et al., 2019; Pike et al., 2020). The availability of genome‐wide data for a large, range‐wide sample of red spruce populations was thus a significant resource to help define an appropriate region for admixture provenancings. From previous work by our team, we knew the southern range edge was genetically distinct from the rest of the species range (Capblancq et al., 2020). Furthermore, because the southern range edge has distinct patterns of genetic diversity as well as unique climate adaptations in both genomics and phenotypic traits (Prakash et al., 2022; Capblancq et al., 2023), it is recommended to conduct seed source provenancing within the region (Lachmuth et al., 2024). By focusing within the range edge for our provenancing strategy, mixing seeds from different populations helped increase the genetic variation while maintaining regional identity to help protect the historical genetic patterns across the landscape. Additionally, the seeds sourced were mostly within the same climate‐based seed zone or were one seed zone away from the restoration site (Pike et al., 2020), which is consistent with the idea of environmental similarity during regional admixture provenancing (sensu Bucharova et al., 2019) (Appendix S5).

**Figure 7:**
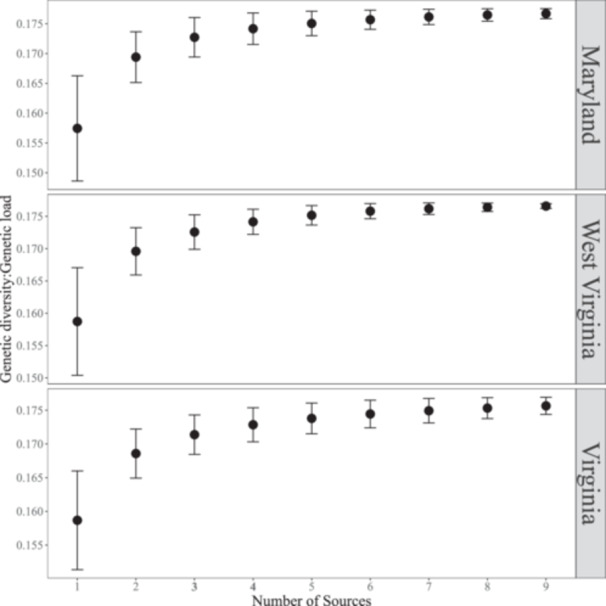
Total number of sources selected for restoration and their respective GD:GL estimates. The estimate for GD:GL flattens out around four to five sources, with diminishing improvement as the number of sources increases.

The number of source sites used in reforestation has been reported to have a strong positive association with GD (Jordan et al., 2019). For our study of red spruce, this raises the question: How many sources should be considered when conducting regional admixture pooling to sufficiently capture the regional genetic diversity present in the southern range edge? In a post‐hoc analysis, we found that GD:GL increased with set size up to around four to five sources, beyond which there were minimal gains in diversity by including additional sources (Figure 7). This analysis was performed after our initial decision to use only three or four sources, which was intended to avoid using too few sources while minimizing practical burden. Moving forward, we would recommend regional admixture provenancing for red spruce consisting of four to five sources to improve the genetic stock of the reforested habitats/region at the range edge. Finally, this approach can also be used to guide seed collection programs to collect and store seeds from a larger number of sources over longer time periods to address the issues associated with periodic cone crops and help build genetic diversity for future climate resiliency.

### Challenges encountered

Procuring high‐quality seeds for red spruce restoration at the landscape level is always challenging because abundant seed production occurs only every 3–8 years (White and Cogbill, 1992), the mature cones stay on the trees for no longer than a year after ripening (Morgenstern and Farrar, 1964), and seed dispersal does not extend beyond 100 m from the mother tree (Hughes and Bechtel, 1997). Although we applied genomic data to identify an optimum seed combination for each site, the final selection of sources was determined by which sources were producing seeds at the time of procurement. Logistical challenges to collecting red spruce cones for reforestation include the need for large, mature trees that ideally occur together in a stand dominated by red spruce so that there is an abundant source of pollen. Additionally, the site should be easily accessible so that field crews can get to the trees with the equipment needed to collect cones from the upper canopy and then transport large numbers of cones back out (red spruce cones weigh ~35 lb/bushel; roughly 1 kg/L). For these reasons, not all the top GD:GL combination sets were logistically feasible for cone collecting. As one example, many of the top GD:GL combination sets for the MD site contained the population GLD (Glades, Maryland). This population is located on a small and difficult‐to‐access parcel surrounded by private landowners with access via right‐of‐ways that are distant from the closest parking. Furthermore, initial scouting trips during autumn 2019 indicated a low cone crop (D. Saville, personal observation). Based on these challenges, we decided to prioritize other sites with better cone crops and easier sampling access. Another challenge is the longer timeline of the scientific investigation, which is not necessarily compatible with the needs of the restoration practices. After our initial source combination model was used to guide the selection of seed provenances in this study, additional research on adaptation in red spruce has been published that helps refine our view in ways relevant to conservation and restoration practice. For example, we have conducted trait‐based and genomic studies of climate adaptation that have helped identify bud phenology traits and abiotic stress response genes that show adaptive signatures to climate across the range of red spruce and that show strong genetic differentiation between the southern range edge and the northern range core (Capblancq et al., 2023). These findings reinforce the decision to maintain a regional scope for the admixture provenancing in the central Appalachians to maintain the locally adapted gene pool, but also point to interesting future directions for sourcing seed provenances for assisted migration northward under a warming climate (Lachmuth et al., 2024).

There were many challenges to the establishment and monitoring of red spruce at a landscape level, as one might expect. However, this work sets up a blueprint for successful collaboration between science and practice with a novel approach using forest genetics for ecological restoration. This combination of science, practice, and monitoring should be seen as a feedback loop rather than a one‐directional transfer of knowledge and skills. The work presented here is just a snapshot in time, marking the beginning of conservation efforts that will hopefully persist for centuries to come.

## AUTHOR CONTRIBUTIONS

A.P., T.C., K.S., and S.K. conceptualized the research idea. A.P., K.S., and S.K. acquired the funding. The methodology was developed by T.C. and S.K. A.P. carried out the curation and visualization of data, and A.P., T.C., and S.K. carried out the formal analysis of the data. K.S. and S.K. were the project administrators, K.S., D.S., and S.K. were responsible for resource acquisition, and K.S., D.S., D.L., C.L., T.J., and S.K. supervised the project. A.P. and S.K. wrote the original draft of the manuscript, and A.P., T.C., K.S., D.S., D.L., C.L., T.J., and S.K. reviewed and edited the manuscript. All authors approved the final version of the manuscript.

## Supporting information


**Appendix S1**. Number of individuals in 23 populations from the southern edge of the red spruce range. This is a subset of the data published in Capblancq et al. (2020) and Prakash et al. (2022).
**Appendix S2**. Marginal effects estimated from a multivariate linear model for early‐life fitness for genetic diversity and genetic load. Recreated from the results of Capblancq et al. (2021) for the red spruce range edge.
**Appendix S3**. Climate PCA for the experimental plots at each restoration site based on climateNA variables (https://climatena.ca/).
**Appendix S4**. Evolvability (*CV*_G_) for phenological traits and height after one year of growth measured for plants raised in a Maryland common garden experiment conducted by Prakash et al. (2022). The traits with the highest *CV*_G_ are represented by gray‐shaded cells. Based on broad sense *H* ^2^ estimates.
**Appendix S5**. The location of red spruce restoration sites and the seed sources selected for planting sites in Maryland, West Virginia, and Virginia. The dark red outlines on the map show the known range extent of red spruce (Little Jr., 1971). The map coloring is based on the Eastern Seed Zone database (Pike et al., 2020).

**Video S1**. Video summarizing the partnership between The Nature Conservancy and the Central Appalachian Spruce Restoration Initiative to restore red spruce forests in the central Appalachians. Video provided courtesy of The Nature Conservancy.

## Data Availability

Annotated code for calculating GD:GL on *n* source population sets, finding optimal combination sets, and analysis of survivorship, growth, and evolvability from field experimental plots is available as Q‐markdown files on GitHub (https://anoobvinu07.github.io/Genomic_assisted_selection/ and the associated repository https://github.com/anoobvinu07/Genomic_assisted_selection) and Zenodo (doi:10.5281/zenodo.10257612).

## References

[aps311600-bib-0001] Allendorf, F. W. 1986. Genetic drift and the loss of alleles versus heterozygosity. Zoo Biology 5(2): 181–190. 10.1002/zoo.1430050212

[aps311600-bib-0002] Bates, D. , M. Mächler , B. Bolker , and S. Walker . 2015. Fitting linear mixed‐effects models using lme4. Journal of Statistical Software 67(1): 1–48. 10.18637/jss.v067.i01

[aps311600-bib-0003] Bengtsson, H. 2023. R.utils: Various Programming Utilities (2.12.3) [Computer software]. http://cran.fhcrc.org/web/packages/R.utils/

[aps311600-bib-0004] Bijlsma, R. , and V. Loeschcke . 2012. Genetic erosion impedes adaptive responses to stressful environments. Evolutionary Applications 5(2): 117–129. 10.1111/j.1752-4571.2011.00214.x 25568035 PMC3353342

[aps311600-bib-0005] Blum, B. M. 1990. *Picea rubens* Sarg. Red spruce. *In* R. M. Burns and B. H. Honkala [eds.], Silvics of North America, 1, 250–259. United States Forest Service, Washington, D.C., USA.

[aps311600-bib-0006] Brancalion, P. H. , and J. van Melis . 2017. On the need for innovation in ecological restoration. Annals of the Missouri Botanical Garden 102(2): 227–236.

[aps311600-bib-0007] Breed, M. F. , P. A. Harrison , C. Blyth , M. Byrne , V. Gaget , N. J. C. Gellie , S. V. C. Groom , et al. 2019. The potential of genomics for restoring ecosystems and biodiversity. Nature Reviews Genetics 20(10): 10. 10.1038/s41576-019-0152-0 31300751

[aps311600-bib-0008] Brubaker, L. B. 1986. Responses of tree populations to climatic change. Vegetatio 67(2): 119–130.

[aps311600-bib-0009] Bucharova, A. , O. Bossdorf , N. Hölzel , J. Kollmann , R. Prasse , and W. Durka . 2019. Mix and match: Regional admixture provenancing strikes a balance among different seed‐sourcing strategies for ecological restoration. Conservation Genetics 20(1): 7–17. 10.1007/s10592-018-1067-6

[aps311600-bib-0010] Burney, O. T. , and D. F. Jacobs . 2011. Ungulate herbivory of regenerating conifers in relation to foliar nutrition and terpenoid production. Forest Ecology and Management 262(9): 1834–1845.

[aps311600-bib-0011] Capblancq, T. , J. R. Butnor , S. Deyoung , E. Thibault , H. Munson , D. M. Nelson , M. C. Fitzpatrick , and S. R. Keller . 2020. Whole‐exome sequencing reveals a long‐term decline in effective population size of red spruce (*Picea rubens*). Evolutionary Applications 13(9): 2190–2205. 10.1111/eva.12985 33005218 PMC7513712

[aps311600-bib-0012] Capblancq, T. , H. Munson , J. R. Butnor , and S. R. Keller . 2021. Genomic drivers of early‐life fitness in *Picea rubens* . Conservation Genetics 22: 963–976. 10.1007/s10592-021-01378-7

[aps311600-bib-0013] Capblancq, T. , S. Lachmuth , M. C. Fitzpatrick , and S. R. Keller . 2023. From common gardens to candidate genes: Exploring local adaptation to climate in red spruce. New Phytologist 237(5): 1590–1605. 10.1111/nph.18465 36068997 PMC10092705

[aps311600-bib-0014] Cingolani, P. , A. Platts , L. L. Wang , M. Coon , T. Nguyen , L. Wang , S. J. Land , X. Lu , and D. M. Ruden . 2012. A program for annotating and predicting the effects of single nucleotide polymorphisms, SnpEff: SNPs in the genome of *Drosophila melanogaster* strain w^1118^; iso‐2; iso‐3. Fly 6(2): 80–92. 10.4161/fly.19695 22728672 PMC3679285

[aps311600-bib-0015] Dumais, D. , and M. Prévost . 2007. Management for red spruce conservation in Québec: The importance of some physiological and ecological characteristics – A review. The Forestry Chronicle 83(3): 378–391. 10.5558/tfc83378-3

[aps311600-bib-0016] Dumais, D. , and M. Prévost . 2016. Germination and establishment of natural red spruce (*Picea rubens*) seedlings in silvicultural gaps of different sizes. The Forestry Chronicle 92(1): 90–100.

[aps311600-bib-0017] Dumais, D. , C. Larouche , P. Raymond , S. Bédard , and M.‐C. Lambert . 2019. Survival and growth dynamics of red spruce seedlings planted under different forest cover densities and types. New Forests 50(4): 573–592. 10.1007/s11056-018-9680-2

[aps311600-bib-0018] Eckert, C. G. , K. E. Samis , and S. C. Lougheed . 2008. Genetic variation across species' geographical ranges: The central–marginal hypothesis and beyond. Molecular Ecology 17(5): 1170–1188. 10.1111/j.1365-294X.2007.03659.x 18302683

[aps311600-bib-0019] ESRI . 2011. ArcGIS desktop: Release 10. Environmental Systems Research Institute, Redlands, California, USA.

[aps311600-bib-0020] Foden, W. B. , B. E. Young , H. R. Akçakaya , R. A. Garcia , A. A. Hoffmann , B. A. Stein , C. D. Thomas , et al. 2019. Climate change vulnerability assessment of species. WIREs Climate Change 10(1): e551. 10.1002/wcc.551

[aps311600-bib-0021] Frankham, R. , K. Lees , M. E. Montgomery , P. R. England , E. H. Lowe , and D. A. Briscoe . 1999. Do population size bottlenecks reduce evolutionary potential? Animal Conservation 2(4): 255–260. 10.1111/j.1469-1795.1999.tb00071.x

[aps311600-bib-0022] Frankham, R. , C. J. A. Bradshaw , and B. W. Brook . 2014. Genetics in conservation management: Revised recommendations for the 50/500 rules, Red List criteria and population viability analyses. Biological Conservation 170: 56–63. 10.1016/j.biocon.2013.12.036

[aps311600-bib-0023] Frankham, R. , J. D. Ballou , K. Ralls , M. D. B. Eldridge , M. R. Dudash , C. B. Fenster , R. C. Lacy , and P. Sunnucks . 2017. Genetic management of fragmented animal and plant populations. Oxford University Press, Oxford, United Kingdom.

[aps311600-bib-0024] García‐Dorado, A. , and A. Caballero . 2021. Neutral genetic diversity as a useful tool for conservation biology. Conservation Genetics 22(4): 541–545. 10.1007/s10592-021-01384-9

[aps311600-bib-0025] Gill, R. M. A. 1992. A review of damage by mammals in north temperate forests: 3. Impact on trees and forests. Forestry: An International Journal of Forest Research 65(4): 363–388. 10.1093/forestry/65.4.363-a

[aps311600-bib-0026] González, A. V. , V. Gómez‐Silva , M. J. Ramírez , and F. E. Fontúrbel . 2020. Meta‐analysis of the differential effects of habitat fragmentation and degradation on plant genetic diversity. Conservation Biology 34(3): 711–720. 10.1111/cobi.13422 31605401

[aps311600-bib-0027] Hadfield, J. D. 2010. MCMC methods for multi‐response generalized linear mixed models: The MCMCglmm R package. Journal of Statistical Software 33(2): 1–22.20808728

[aps311600-bib-0028] Hoban, S. , M. W. Bruford , W. C. Funk , P. Galbusera , M. P. Griffith , C. E. Grueber , M. Heuertz , et al. 2021. Global commitments to conserving and monitoring genetic diversity are now necessary and feasible. Bioscience 71(9): 964–976. 34475806 10.1093/biosci/biab054 PMC8407967

[aps311600-bib-0029] Hoffmann, A. A. , A. D. Miller , and A. R. Weeks . 2021. Genetic mixing for population management: From genetic rescue to provenancing. Evolutionary Applications 14(3): 634–652. 10.1111/eva.13154 33767740 PMC7980264

[aps311600-bib-0030] Houle, D. 1992. Comparing evolvability and variability of quantitative traits. Genetics 130(1): 195–204. 10.1093/genetics/130.1.195 1732160 PMC1204793

[aps311600-bib-0031] Hufford, K. M. , and S. J. Mazer . 2003. Plant ecotypes: Genetic differentiation in the age of ecological restoration. Trends in Ecology & Evolution 18(3): 147–155. 10.1016/S0169-5347(03)00002-8

[aps311600-bib-0032] Hughes, J. W. , and D. A. Bechtel . 1997. Effect of distance from forest edge on regeneration of red spruce and balsam fir in clearcuts. Canadian Journal of Forest Research 27(12): 2088–2096.

[aps311600-bib-0033] Jordan, R. , M. F. Breed , S. M. Prober , A. D. Miller , and A. A. Hoffmann . 2019. How well do revegetation plantings capture genetic diversity? Biology Letters 15(10): 20190460. 10.1098/rsbl.2019.0460 31615374 PMC6832181

[aps311600-bib-0034] Kimmins, J. , and D. Lavender . 1987. Implications of climate change for the distribution of biogeoclimatic zones in British Columbia and for the growth of temperate forest species. *In* D. P. Lavender [ed.], Woody plant growth in a changing chemical and physical environment, 209–309. University of British Columbia Press, Vancouver, Canada.

[aps311600-bib-0035] Kitzmiller, J. H. 1990. Managing genetic diversity in a tree improvement program. Forest Ecology and Management 35(1): 131–149. 10.1016/0378-1127(90)90237-6

[aps311600-bib-0036] Korneliussen, T. S. , A. Albrechtsen , and R. Nielsen . 2014. ANGSD: Analysis of Next Generation Sequencing Data. BMC Bioinformatics 15(1): 1–13. 25420514 10.1186/s12859-014-0356-4 PMC4248462

[aps311600-bib-0037] Korstian, C. F. 1937. Perpetuation of spruce on cut‐over and burned lands in the higher southern Appalachian Mountains. Ecological Monographs 7(1): 125–167.

[aps311600-bib-0038] Lachmuth, S. , T. Capblancq , A. Prakash , S. R. Keller , and M. C. Fitzpatrick . 2024. Novel genomic offset metrics integrate local adaptation into habitat suitability forecasts and inform assisted migration. Ecological Monographs 94(1): e1593. 10.1002/ecm.1593

[aps311600-bib-0039] Leberg, P. L. 1992. Effects of population bottlenecks on genetic diversity as measured by allozyme electrophoresis. Evolution 46(2): 477–494. 10.1111/j.1558-5646.1992.tb02053.x 28564024

[aps311600-bib-0040] Little, Jr., E. L. 1971. Atlas of United States trees, vol. 1. Conifers and important hardwoods. USDA Forest Service Miscellaneous Publication 1146. U.S. Department of Agriculture, U.S. Forest Service, Washington, D.C., USA.

[aps311600-bib-0041] Mathias, J. M. , and R. B. Thomas . 2018. Disentangling the effects of acidic air pollution, atmospheric CO_2_, and climate change on recent growth of red spruce trees in the Central Appalachian Mountains. Global Change Biology 24(9): 3938–3953. 10.1111/gcb.14273 29781219

[aps311600-bib-0042] McCreary, D. , D. Lavender , and R. Hermann . 1990. Predicted global warming and Douglas‐fir chilling requirements. Annales Des Sciences Forestières 47(4): 325–330. 10.1051/forest:19900404

[aps311600-bib-0043] Morgenstern, E. K. , and J. L. Farrar . 1964. Introgressive hybridization in Red Spruce and Black Spruce. Technical Report, Faculty of Forestry, University of Toronto, no. 4. University of Toronto, Toronto, Canada.

[aps311600-bib-0044] Nei, M. 1973. Analysis of gene diversity in subdivided populations. Proceedings of the National Academy of Sciences, USA 70(12): 3321–3323. 10.1073/pnas.70.12.3321 PMC427228 4519626

[aps311600-bib-0045] Nei, M. , T. Maruyama , and R. Chakraborty . 1975. The bottleneck effect and genetic variability in populations. Evolution 29(1): 1–10. 10.2307/2407137 28563291

[aps311600-bib-0046] Nystedt, B. , N. R. Street , A. Wetterbom , A. Zuccolo , Y.‐C. Lin , D. G. Scofield , F. Vezzi , et al. 2013. The Norway spruce genome sequence and conifer genome evolution. Nature 497(7451): 579–584. 23698360 10.1038/nature12211

[aps311600-bib-0047] Ørsted, M. , A. A. Hoffmann , E. Sverrisdóttir , K. L. Nielsen , and T. N. Kristensen . 2019. Genomic variation predicts adaptive evolutionary responses better than population bottleneck history. PLoS Genetics 15(6): e1008205. 10.1371/journal.pgen.1008205 31188830 PMC6590832

[aps311600-bib-0048] Pachauri, R. K. , M. R. Allen , V. R. Barros , J. Broome , W. Cramer , R. Christ , J. A. Church , et al. 2014. Climate change 2014: Synthesis report. Contribution of Working Groups I, II and III to the fifth assessment report of the Intergovernmental Panel on Climate Change. IPCC, Geneva, Switzerland. https://www.ipcc.ch/site/assets/uploads/2018/02/SYR_AR5_FINAL_full.pdf

[aps311600-bib-0049] Parmesan, C. , and G. Yohe . 2003. A globally coherent fingerprint of climate change impacts across natural systems. Nature 421(6918): 37–42. 10.1038/nature01286 12511946

[aps311600-bib-0050] Pecl, G. T. , M. B. Araújo , J. D. Bell , J. Blanchard , T. C. Bonebrake , I.‐C. Chen , T. D. Clark , et al. 2017. Biodiversity redistribution under climate change: Impacts on ecosystems and human well‐being. Science 355(6332): eaai9214. 10.1126/science.aai9214 28360268

[aps311600-bib-0051] Pike, C. , K. M. Potter , P. Berrang , B. Crane , J. Baggs , L. Leites , and T. Luther . 2020. New seed‐collection zones for the eastern United States: The Eastern Seed Zone Forum. Journal of Forestry 118(4): 444–451.

[aps311600-bib-0052] Prakash, A. , S. DeYoung , S. Lachmuth , J. L. Adams , K. Johnsen , J. R. Butnor , D. M. Nelson , et al. 2022. Genotypic variation and plasticity in climate‐adaptive traits after range expansion and fragmentation of red spruce (*Picea rubens* Sarg.). Philosophical Transactions of the Royal Society B: Biological Sciences 377(1848): 20210008. 10.1098/rstb.2021.0008 PMC8859516 35184589

[aps311600-bib-0053] Prober, S. M. , M. Byrne , E. H. McLean , D. A. Steane , B. M. Potts , R. E. Vaillancourt , and W. D. Stock . 2015. Climate‐adjusted provenancing: A strategy for climate‐resilient ecological restoration. Frontiers in Ecology and Evolution 3: 65.

[aps311600-bib-0054] Ralls, K. , P. Sunnucks , R. C., Lacy , and R. Frankham . 2020. Genetic rescue: A critique of the evidence supports maximizing genetic diversity rather than minimizing the introduction of putatively harmful genetic variation. Biological Conservation 251: 108784. 10.1016/j.biocon.2020.108784

[aps311600-bib-0055] Raymond, P. , and S. Bédard . 2017. The irregular shelterwood system as an alternative to clearcutting to achieve compositional and structural objectives in temperate mixedwood stands. Forest Ecology and Management 398: 91–100.

[aps311600-bib-0056] Reams, G. A. , and P. C. V. Deusen . 1993. Synchronie large‐scale disturbances and red spruce growth decline. Canadian Journal of Forest Research 23(7): 1361–1374.

[aps311600-bib-0057] Reed, D. H. , and R. Frankham . 2003. Correlation between fitness and genetic diversity. Conservation Biology 17(1): 230–237. 10.1046/j.1523-1739.2003.01236.x

[aps311600-bib-0058] Schindler, D. E. , J. B. Armstrong , and T. E. Reed . 2015. The portfolio concept in ecology and evolution. Frontiers in Ecology and the Environment 13(5): 257–263. 10.1890/140275

[aps311600-bib-0059] Schopmeyer, C. S. 1974. Seeds of woody plants in the United States. U.S. Department of Agriculture, Washington, D.C., USA.

[aps311600-bib-0060] Sork, V. L. , F. W. Davis , P. E. Smouse , V. J. Apsit , R. J. Dyer , J. F. Fernandez‐M , and B. Kuhn . 2002. Pollen movement in declining populations of California Valley oak, *Quercus lobata*: Where have all the fathers gone? Molecular Ecology 11(9): 1657–1668. 10.1046/j.1365-294x.2002.01574.x 12207717

[aps311600-bib-0061] Suding, K. , E. Higgs , M. Palmer , J. B. Callicott , C. B. Anderson , M. Baker , J. J. Gutrich , et al. 2015. Committing to ecological restoration. Science 348(6235): 638–640. 10.1126/science.aaa4216 25953995

[aps311600-bib-0062] Teixeira, J. C. , and C. D. Huber . 2021. The inflated significance of neutral genetic diversity in conservation genetics. Proceedings of the National Academy of Sciences, USA 118(10): e2015096118. 10.1073/pnas.2015096118 PMC7958437 33608481

[aps311600-bib-0063] Theissinger, K. , C. Fernandes , G. Formenti , I. Bista , P. R. Berg , C. Bleidorn , A. Bombarely , et al. 2023. How genomics can help biodiversity conservation. Trends in Genetics 39(7): 545–559. 10.1016/j.tig.2023.01.005 36801111

[aps311600-bib-0064] Tukey, J. W. 1977. Exploratory data analysis, 2. Addison‐Wesley, Reading, Massachusetts, USA.

[aps311600-bib-0065] USDA . 2021. The PLANTS Database. National Plant Data Team, Greensboro, North Carolina, USA. Website: http://plants.usda.gov [accessed 13 May 2024].

[aps311600-bib-0066] Vakkari, P. , A. Blom , M. Rusanen , J. Raisio , and H. Toivonen . 2006. Genetic variability of fragmented stands of pedunculate oak (*Quercus robur*) in Finland. Genetica 127(1): 231–241. 10.1007/s10709-005-4014-7 16850227

[aps311600-bib-0067] Vranckx, G. , H. Jacquemyn , B. Muys , and O. Honnay . 2012. Meta‐analysis of susceptibility of woody plants to loss of genetic diversity through habitat fragmentation. Conservation Biology 26(2): 228–237. 10.1111/j.1523-1739.2011.01778.x 22044646

[aps311600-bib-0068] White, P. S. , and C. V. Cogbill . 1992. Spruce‐fir forests of eastern North America. *In* Ecology and decline of red spruce in the eastern United States, 3–39. Springer, New York, New York, USA.

[aps311600-bib-0069] Willi, Y. , M. Fracassetti , S. Zoller , and J. Van Buskirk . 2018. Accumulation of mutational load at the edges of a species range. Molecular Biology and Evolution 35(4): 781–791. 10.1093/molbev/msy003 29346601

[aps311600-bib-0070] Willi, Y. , T. N. Kristensen , C. M. Sgrò , A. R. Weeks , M. Ørsted , and A. A. Hoffmann . 2022. Conservation genetics as a management tool: The five best‐supported paradigms to assist the management of threatened species. Proceedings of the National Academy of Sciences, USA 119(1): e2105076119. 10.1073/pnas.2105076119 PMC8740573 34930821

[aps311600-bib-0071] Woodruff, D. S. 2001. Populations, species, and conservation genetics. Encyclopedia of Biodiversity 2001: 811.

[aps311600-bib-0072] Wu, X. , J. F. McCormick , and R. T. Busing . 1999. Growth pattern of *Picea rubens* prior to canopy recruitment. Plant Ecology 140: 245–253.

