# Supplementary material for: Bringing genomics to the field: An integrative approach to seed sourcing for forest restoration
Source: Appl Plant Sci. 2024 Jun 20;12(3):e11600. doi: 10.1002/aps3.11600 (PMC11192164; doi:10.1002/aps3.11600)
Supplement: Supplementary file 1 — Appendix S1. Number of individuals in 23 populations from the southern edge of the red spruce range. This is a subset of the data published in Capblancq et al. (2020) and Prakash et al. (2022). Appendix S2. Marginal effects estimated from a multivariate linear model for early‐life fitness for genetic diversity and genetic load. Recreated from the results of Capblancq et al. (2021) for the red spruce range edge. Appendix S3. Climate PCA for the experimental plots at each restoration site based on climateNA variables (https://climatena.ca/). Appendix S4. Evolvability (CV G) for phenological traits and height after one year of growth measured for plants raised in a Maryland common garden experiment conducted by Prakash et al. (2022). The traits with the highest CV G are represented by gray‐shaded cells. Based on broad sense H 2 estimates. Appendix S5. The location of red spruce restoration sites and the seed sources selected for planting sites in Maryland, West Virginia, and Virginia. The dark red outlines on the map show the known range extent of red spruce (Little Jr., 1971). The map coloring is based on the Eastern Seed Zone database (Pike et al., 2020). [file APS3-12-e11600-s001.docx]

**Supplementary appendices for “Bringing genomics to the field: An integrative approach to seed sourcing for forest restoration.”**


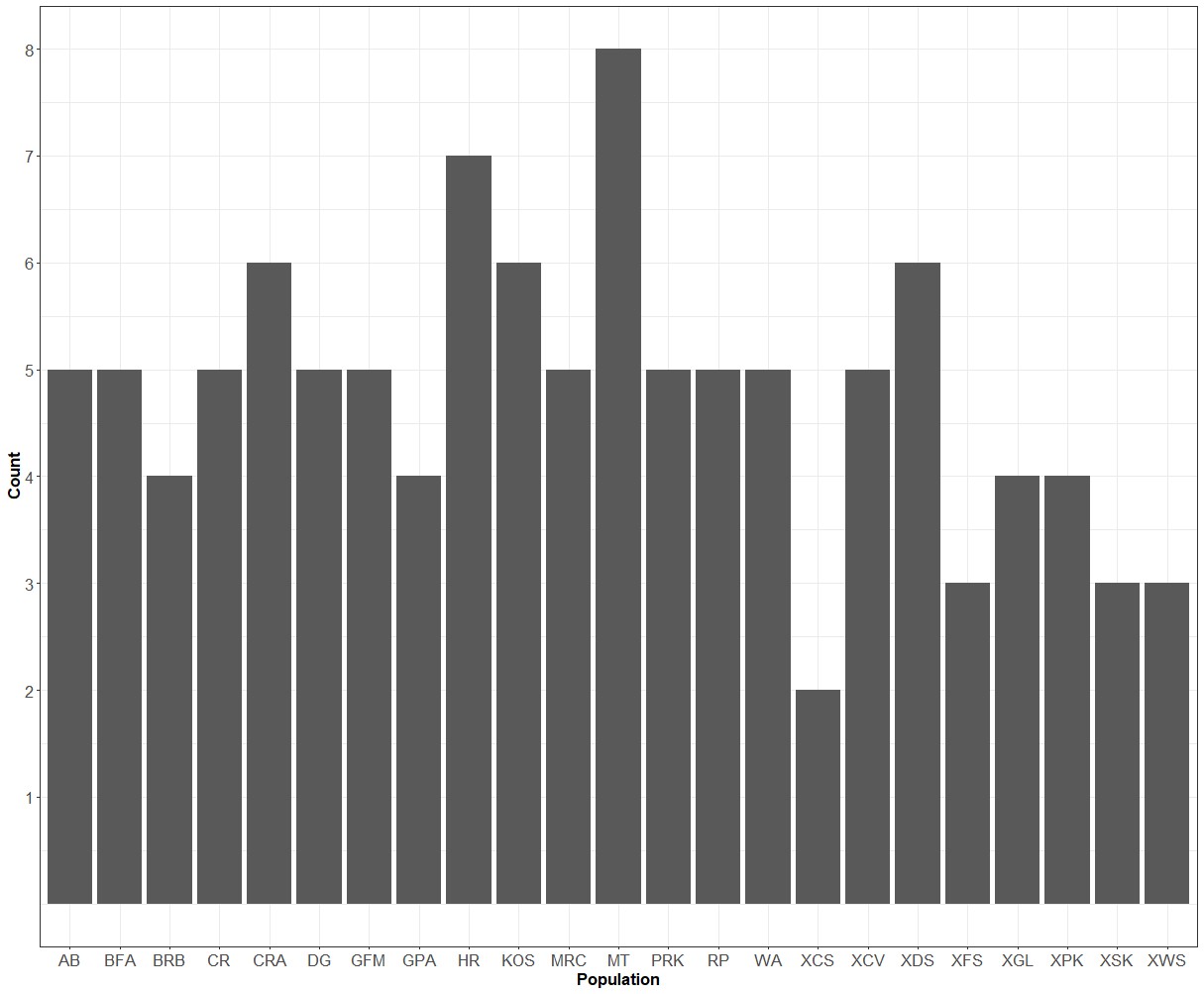


**Appendix S1.** Number of individuals in 23 populations from the southern edge of the red spruce range. This is a subset of the data published in Capblancq et al. (2020) and Prakash et al. (2022).


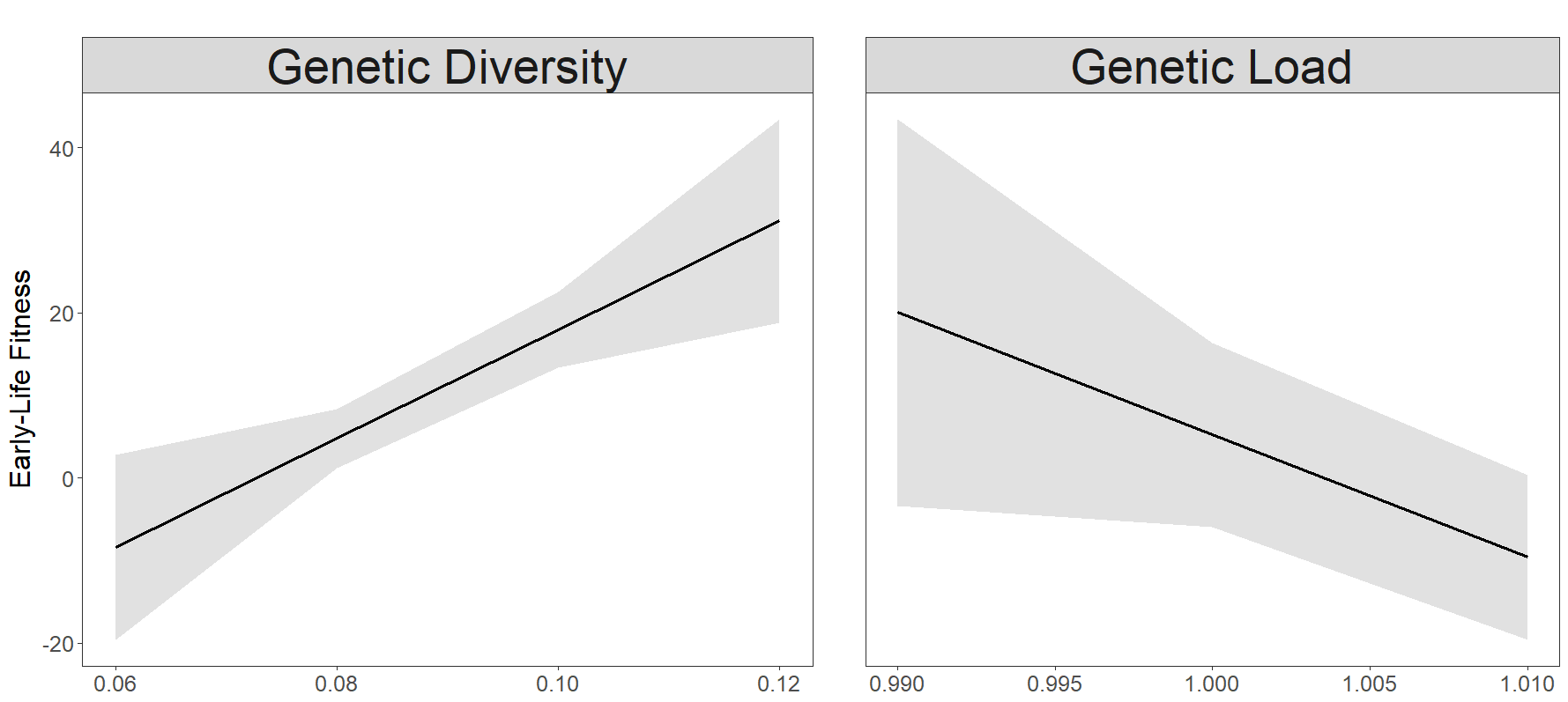


**Appendix S2.** Marginal effects estimated from a multivariate linear model for early-life fitness for genetic diversity and genetic load. Recreated from the results of Capblancq et al. (2021) for the red spruce range edge.


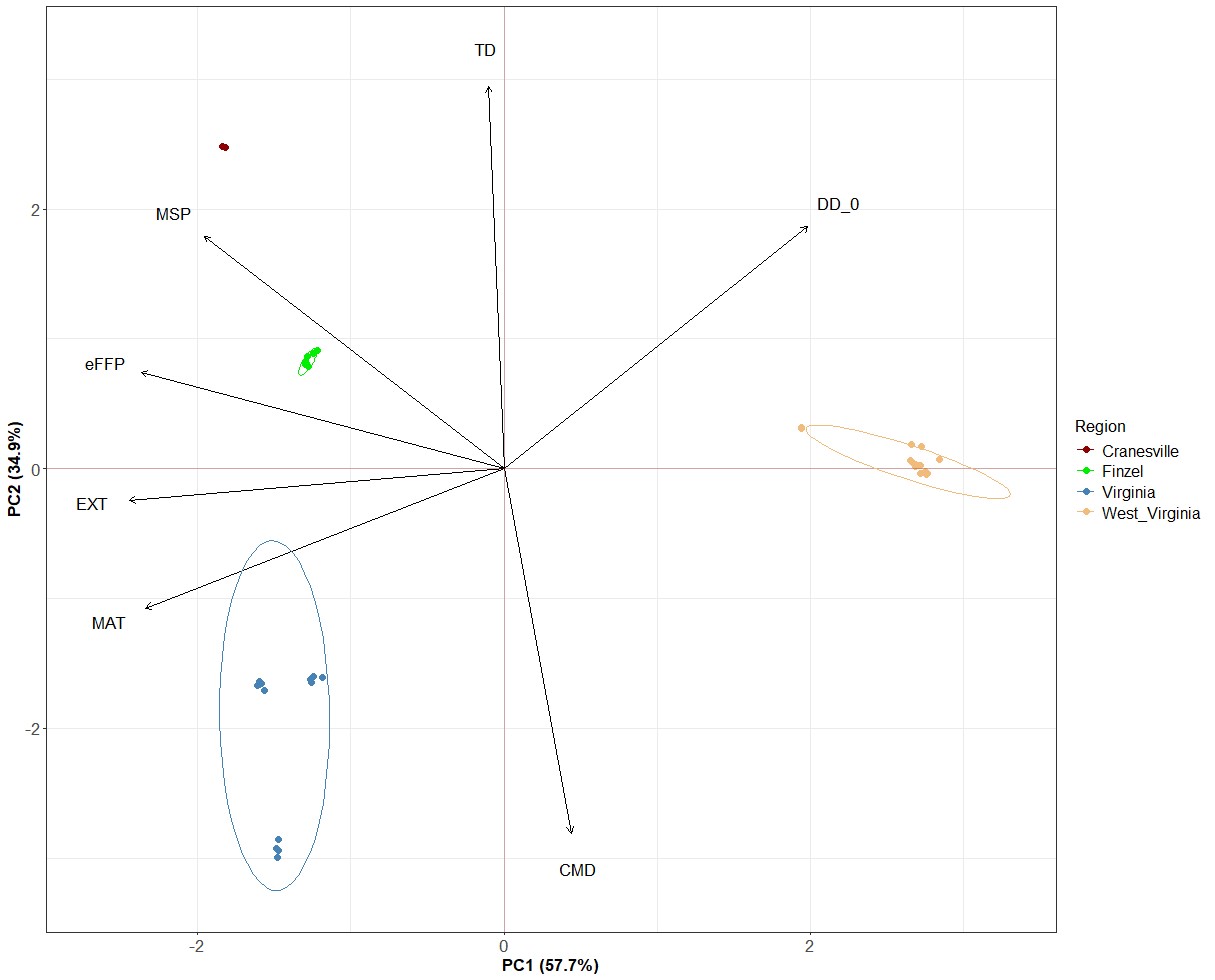


**Appendix S3.** Climate PCA for the experimental plots at each restoration site based on climateNA variables (<https://climatena.ca/>). CMD = Hargreaves climatic moisture deficit (mm), eFFP = the day of the year on which frost-free period ends, EXT = extreme maximum temperature over 30 years, DD_0 = degree-days below 0°C, MAT = mean annual temperature (°C), MSP = May to September precipitation (mm), TD = continentality (°C).

**Appendix S4.** Evolvability (*CV*_G_) for phenological traits and height after one year of growth measured for plants raised in a Maryland common garden experiment conducted by Prakash et al. (2022). The traits with the highest *CV*_G_ are represented by gray-shaded cells. Based on broad sense *H*^2^ estimates.^a^

| **Trait** | $\bar{\boldsymbol{X}}$ | ***V*_P_** | ***H*^2^** | ***V*_G_** | ***CV*_G_** |
| --- | --- | --- | --- | --- | --- |
| Height | 31.1266 | 8.2665 | 0.3962 | 3.2751 | 0.0581 |
| Bud break (2020) | 675.465 | 17.9483 | 0.0274 | 0.4916 | 0.001 |
| Bud set (2019) | 261.6267 | 17.8719 | 0.1677 | 2.9968 | 0.0066 |
| Bud set (2020) | 209.9607 | 1.0749 | 0.017 | 0.0183 | 0.0006 |

*Note:* $\bar{X}$ $= phenotypic mean, V$_P_ = phenotypic variance, *H^2^* = broad sense heritability, *V*_G_ = genetic variance, *CV*_G_ = coefficient of evolvability.

^a^ Heritability measured for height at the Vermont and North Carolina common gardens are 0.2844 and 0.3251, respectively.


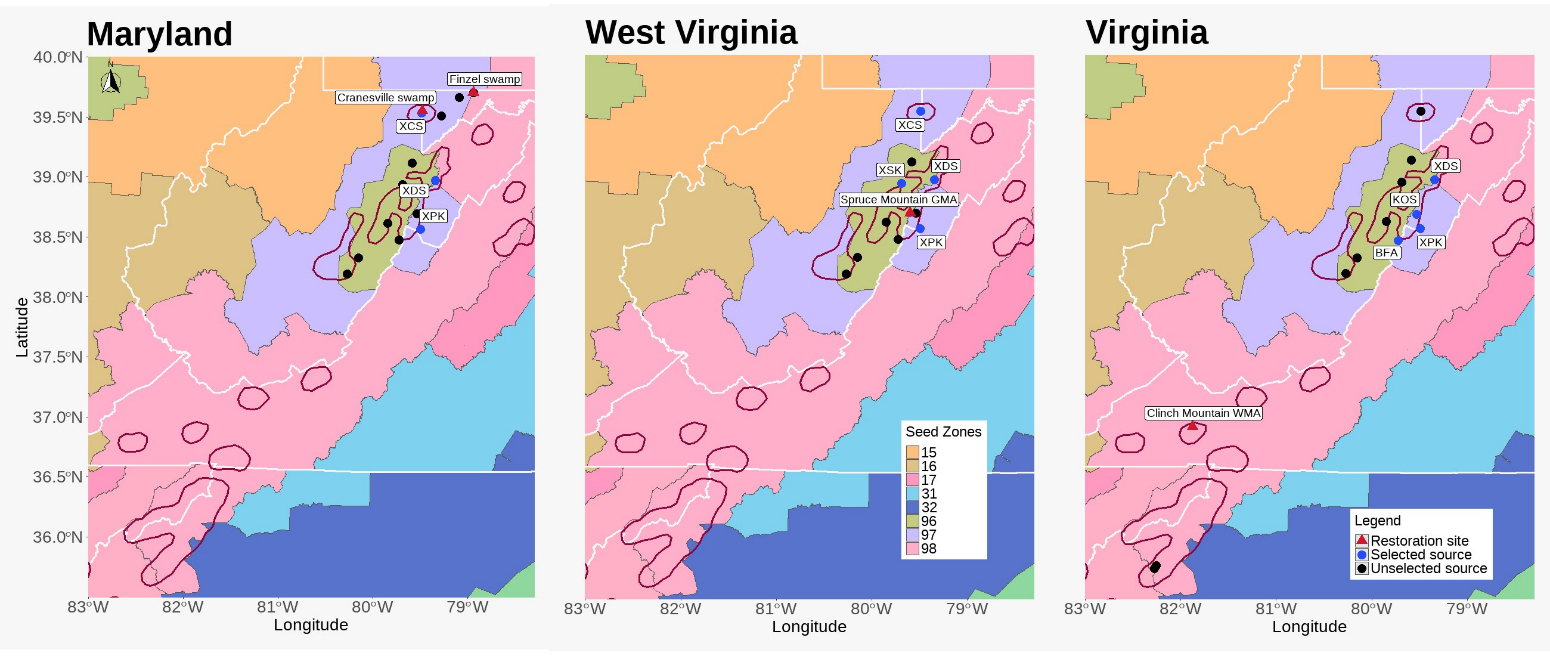


**Appendix S5.** The location of red spruce restoration sites and the seed sources selected for planting sites in Maryland, West Virginia, and Virginia. The dark red outlines on the map show the known range extent of red spruce (Little Jr., 1971). The map coloring is based on the Eastern Seed Zone database (Pike et al., 2020).

**REFERENCES**

Capblancq, T., H. Munson, J. R. Butnor, and S. R. Keller. 2021. Genomic drivers of early-life fitness in *Picea rubens*. *Conservation Genetics* 22: 963–976. <https://doi.org/10.1007/s10592-021-01378-7>

Little Jr., E. L. 1971. Atlas of United States trees, vol. 1. Conifers and important hardwoods. USDA Forest Service Miscellaneous Publication 1146. U.S. Department of Agriculture, U.S. Forest Service, Washington, D.C., USA.

Pike, C., K. M. Potter, P. Berrang, B. Crane, J. Baggs, L. Leites, and T. Luther. 2020. New seed-collection zones for the eastern United States: The Eastern Seed Zone Forum. *Journal of Forestry* 118(4): 444–451.

Prakash, A., S. DeYoung, S. Lachmuth, J. L. Adams, K. Johnsen, J. R. Butnor, D. M. Nelson, et al. 2022. Genotypic variation and plasticity in climate-adaptive traits after range expansion and fragmentation of red spruce (*Picea rubens* Sarg.). *Philosophical Transactions of the Royal Society B: Biological Sciences* 377(1848): 20210008. https://doi.org/10.1098/rstb.2021.0008
